# Inhibitory Effects of Pretreatment with Radon on Acute Alcohol-Induced Hepatopathy in Mice

**DOI:** 10.1155/2012/382801

**Published:** 2012-11-14

**Authors:** Teruaki Toyota, Takahiro Kataoka, Yuichi Nishiyama, Takehito Taguchi, Kiyonori Yamaoka

**Affiliations:** ^1^Graduate School of Health Sciences, Okayama University, 5-1 Shikata-cho, 2-chome, Okayama 700-8558, Japan; ^2^Department of Radiation Research Shikata Laboratory, Advanced Science Research Center, Okayama University, 5-1 Shikata-cho, 2-chome, Okayama 700-8558, Japan

## Abstract

We previously reported that radon inhalation activates antioxidative functions in the liver and inhibits carbon tetrachloride-induced hepatopathy in mice. In addition, it has been reported that reactive oxygen species contribute to alcohol-induced hepatopathy. In this study, we examined the inhibitory effects of radon inhalation on acute alcohol-induced hepatopathy in mice. C57BL/6J mice were subjected to intraperitoneal injection of 50% alcohol (5 g/kg bodyweight) after inhaling approximately 4000 Bq/m^3^ radon for 24 h. Alcohol administration significantly increased the activities of glutamic oxaloacetic transaminase (GOT), glutamic pyruvic transaminase (GPT) in serum, and the levels of triglyceride and lipid peroxide in the liver, suggesting acute alcohol-induced hepatopathy. Radon inhalation activated antioxidative functions in the liver. Furthermore, pretreatment with radon inhibited the depression of hepatic functions and antioxidative functions. These findings suggested that radon inhalation activated antioxidative functions in the liver and inhibited acute alcohol-induced hepatopathy in mice.

## 1. Introduction

Radon is a radioactive gaseous element that mainly emits *α*-rays. On entry through the lungs or skin, it reaches the blood stream and is then distributed throughout the body. If radon is inhaled, the lungs will be subjected to the actions of free radicals created by radiation and may suffer inflammation. Although radon inhalation has been thought to be hazardous in general, a large number of patients are treated in various countries with traditional radon spa therapy (Japan [1–4], central Europe [5]). In particular, therapy involving radon gas volatilized from radon-enriched water is performed for various diseases at Misasa Medical Center, Okayama University Hospital [1, 6]. The most common diseases treated with radon therapy are arteriosclerosis, osteoarthritis [1], and bronchial asthma [6], which are related to reactive oxygen species (ROS). To assess the effects of radon, we developed a radon-exposure system and conducted several studies. For example, in the search for new indications for radon therapy, we reported the responsiveness to radon of an antioxidant enzyme (a radical scavenger), superoxide dismutase (SOD), in mouse organs [7], and suggested that radon inhalation activated antioxidative functions in many organs of mice. In addition, it was demonstrated that radon inhalation has anti-inflammatory effects and inhibits carrageenan-induced inflammatory paw edema [8]. Furthermore, a clinical study was conducted using thoron, which is an isotope of radon. The results suggested that thoron and thermal treatment activate antioxidative functions, and that thoron and thermal treatment prevent diabetic ketoacidosis [3]. Thus, it is likely that radon therapy would be useful for several diseases. In particular, radon therapy is less stressful than other therapies because it is a colorless, tasteless, and odorless gas. On the other hand, several reports have indicated the health risks of high-level radon, especially for the lungs and skin [9–11]; however, in radon therapy at Misasa, therapeutic exposure doses within the range of 50–67 *μ*Sv are lower than the average annual amount of natural radiation (2.4 mSv) [12]. Additionally, according to Sakoda's report [13], the dose absorbed by the lungs was 300 nGy and by the skin was 152 nGy under the conditions of this study.

ROS, such as hydrogen peroxide (H_2_O_2_), hydroxyl radical (^−^OH), and superoxide anion radicals (O_2_^−^), damage DNA, lipids, and enzymes and are highly toxic. Cells can be injured or killed when the ROS level exceeds the cellular antioxidant capacity. For example, there is evidence that oxidative stress plays an important role in the development of alcoholic liver disease [14–17]. Alcohol administration has been found to cause the accumulation of ROS, including O_2_^−^, ^−^OH, and H_2_O_2_, and to increase lipid peroxide levels in the liver. 

Although hepatic damage is not the main indication for radon therapy, our previous study demonstrated that radon inhalation clearly inhibited carbon tetrachloride (CCl_4_)-induced hepatopathy [18]; however, it is unlikely that humans will suffer from CCl_4_-induced hepatopathy. In contrast, alcohol-induced hepatopathy is a common contemporary disease. In our previous study, mice inhaled radon immediately after alcohol intake to demonstrate the relation between alcohol metabolism and the activation of antioxidative functions in the liver and brain by radon inhalation [19]. These findings suggest that radon inhalation activated antioxidative functions in the liver and brain of mice, but the brain was more susceptible to oxidation by radon than the liver.

The purpose of this study was to assess whether pretreatment with radon inhibits acute alcohol-induced hepatopathy in mice. The following biochemical and histological parameters were examined to assess the effects of radon treatment on antioxidative functions: SOD, catalase, total glutathione content (t-GSH), glutathione peroxidase (GPx) and glutathione reductase (GR) activities, lipid peroxide levels in the liver, glutamic oxaloacetic transaminase (GOT), glutamic pyruvic transaminase (GPT), triglyceride level (TG) total cholesterol (T-CHO) in serum, TG in the liver, and histological examination of liver tissue.

## 2. Materials and Methods

### 2.1. Radon Exposure System

**Figure 1:**
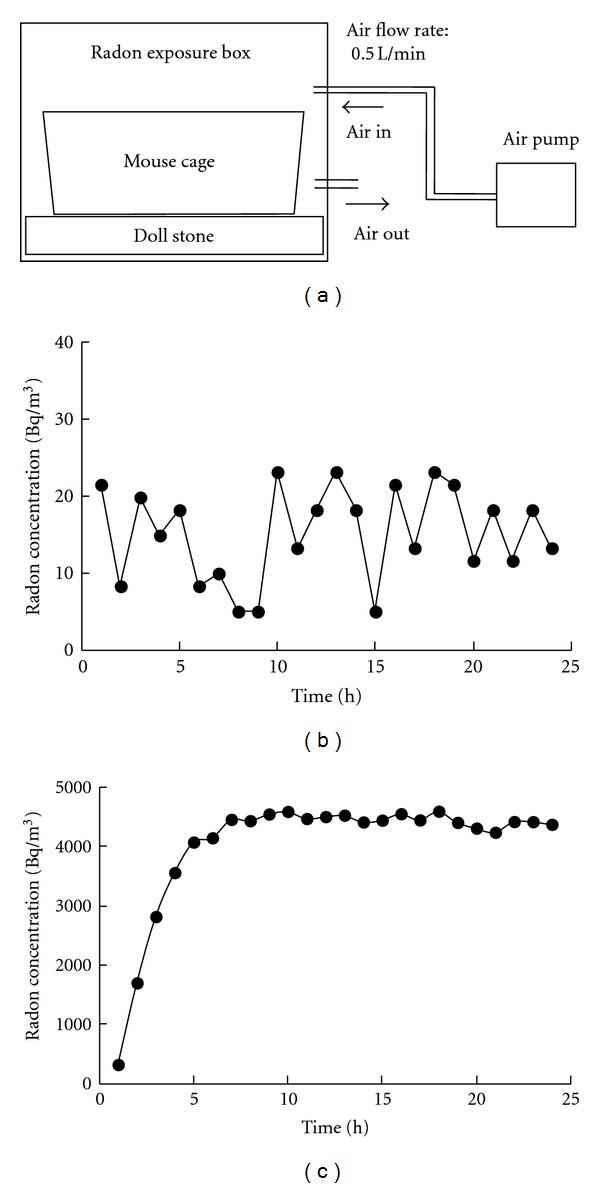
Schematic diagram of the radon exposure system (a). Changes in the radon concentration in the mouse cage over the period of sham (b) or radon inhalation (c).

The radon exposure system is shown in Figure 1(a). Approximately 8000 g of the “Doll Stone” radon source (Ningyotoge Gensiryoku Sangyo, Co., Ltd., Okayama, Japan) was placed under a mouse cage in a radon exposure box (370 mm × 260 mm × 272 mm) to generate the conditions for radon inhalation. Air was blown into the box at a rate of 0.5 L/min using an air pump, and an exhaust vent was opened. Mice had free access to food and water during radon inhalation. The radon concentration in the mouse cage was measured using a radon monitor (CRM-510, Measure Work, Chiba, Japan). The mean radon concentrations were approximately 20 (background) and 4000 Bq/m^3^, respectively.

### 2.2. Experimental Protocol

Male C57BL/6J mice (age: eight weeks; weight: approximately 20 g) were obtained from the Department of Animal Resources Advanced Science Research Center, Okayama University, Okayama, Japan. Ethics approval was obtained from the animal experimental committee of Okayama University. The study protocol was in accordance with the animal experimental guidelines of Okayama University. Each experimental group consisted of 4–6 mice.

Mice inhaled radon at a concentration of 4000 Bq/m^3^ for 24 hours. Then, 5 g/kg bodyweight of alcohol or saline solution (control) was injected into the peritoneum of the mice. Six or twenty-four hours after alcohol administration, blood was drawn from the heart for serum analysis, and livers were quickly excised to analyze the levels of SOD, catalase, t-GSH, GPx, GR, TG, and lipid peroxide. Serum was separated by centrifugation at 3000 ×g for 5 min for assays of GOT and GPT, activity and the TG, T-CHO, and protein levels. These samples were preserved at −80°C until biochemical assay. Liver tissue samples were fixed in 10% neutral-buffered formalin for histological examinations.

### 2.3. Biochemical Assays

The serum activity of GOT and GPT, serum levels of TG and T-CHO, and the levels of TG in the liver were measured using TA-LN kainosu, TG-EN kainosu, and T-CHO kainosu, respectively (Kainosu Co., Ltd., Tokyo, Japan), according to the manufacturer's recommendations. 

The protein content was measured by the Bradford method, using the Protein Quantification Kit-Rapid (Dojindo Molecular Technologies, Inc., Kumamoto, Japan) [20].

Lipid peroxide (malondialdehyde (MDA)) levels were assayed using the Bioxytech LPO-586 assay kit (OXIS Health Products, Inc., Portland, OR, USA). Briefly, livers were homogenized in 20 mM phosphate buffer (PBS; pH 7.4) on ice. Prior to homogenization, 10 *μ*L of 0.5 M butylated hydroxytoluene in acetonitrile were added per 1 mL of tissue homogenate. After homogenization, the homogenate was centrifuged at 15 000 ×g for 10 min at 4°C and the supernatant was used for the assay. The MDA assay is based on the reaction of a chromogenic reagent, N-methyl-2-phenylidole, with MDA at 45°C. The optical density of the colored products was read at 586 nm on a spectrophotometer.

Mouse liver was homogenized in a 1 M Tris-HCl buffer containing 5 mM ethylenediaminetetraacetic acid (EDTA) (pH 7.4) on ice. The homogenate was centrifuged at 12 000 ×g for 45 min at 4°C, and the supernatant was used to assay the activity of SOD and catalase.

SOD activity was measured by the nitroblue tetrazolium (NBT) reduction method [21] using the Wako-SOD test (Wako Pure Chemical Industries, Co., Ltd., Osaka, Japan). Briefly, the extent of inhibition of NBT reduction was measured at 560 nm using a spectrophotometer. One unit of enzyme activity was defined as 50% inhibition of NBT reduction. 

 Catalase activity was measured as the H_2_O_2_ reduction rate at 37°C and was assayed at 240 nm using a spectrophotometer [22]. The assay mixture consisted of 50 *μ*L of 1 M Tris-HCl buffer containing 5 mM EDTA (pH 7.4), 900 *μ*L of 10 mM H_2_O_2_, 30 *μ*L deionized water, and 20 *μ*L liver supernatant. Activity was calculated using a molar extinction coefficient of 7.1 × 10^−3^ M^−1 ^cm^−1^. Catalase activity was measured by the amount of hydrogen peroxide split by catalase in 20, 40, or 60 sec at 37°C. The reactions were started by addition of the liver supernatant.

Total glutathione content was measured using the Bioxytech GSH-420 assay kit (OXIS Health Products). Briefly, livers were homogenized in 10 mM phosphate buffer (pH 7.4) on ice and then mixed with ice-cold 7.5% trichloroacetic acid solution. The homogenates were centrifuged at 3000 ×g for 10 min. The supernatant was used for the assay. Total glutathione content was measured at 420 nm using a spectrophotometer. This assay is based on the formation of a chromophoric thione, the absorbance of which, measured at 420 nm, is directly proportional to the total glutathione concentration. 

GPx activity was measured using the Bioxytech GPx-340 assay kit (OXIS Health Products, Inc.). Briefly, liver samples were homogenized in 1 M Tris-HCl buffer (pH 7.4) containing 5 mM EDTA and 1 mM dithiothreitol. The homogenates were centrifuged at 10 000 ×g for 20 min at 4°C. The supernatants were used for the assay. The reduction of nicotinamide adenine dinucleotide phosphate (NADPH) to nicotinamide adenine dinucleotide phosphate (NADP^+^) is accompanied by a decrease in absorbance, measured at 340 nm, providing a spectrophotometric means for monitoring GPx enzyme activity. The molar extinction coefficient for NADPH is 6220 M^−1 ^cm^−1^ at 340 nm. To assay GPx, the supernatant is added to a solution containing glutathione, GR, and NADPH. The enzyme reaction is initiated by adding a substrate, tert-butyl hydroperoxide, and absorbance at 340 nm is recorded for 3 min.

GR activity was assayed using the Bioxytech GR-340 assay kit (OXIS Health Products, Inc.). Briefly, liver samples were homogenized in 1 M Tris-HCl buffer with 5 mM EDTA acid (pH 7.4) on ice. The homogenates were centrifuged at 8500 ×g for 10 min, and the supernatants were used for the assay. The assay is based on the oxidation of NADPH to NADP^+^, catalyzed by a limiting concentration of GR. One GR activity unit is defined as the amount of enzyme that catalyzes the reduction of 1 *μ*mol of oxidized glutathione (GSSG) per minute at pH 7.4 and 25°C. The reduction of GSSG is determined indirectly by measuring the consumption of NADPH, as demonstrated by a decrease in absorbance at 340 nm as a function of time.

### 2.4. Histological Examination

Liver samples were fixed in 10% formalin, processed through a graded ethanol series and finally xylene, and embedded in paraffin. Six-micrometer-thick tissue sections were prepared and stained with hematoxylin-eosin (HE).

### 2.5. Statistical Analyses

Data are presented as the mean ± standard error of the mean (SEM). Statistically significant differences between two groups were determined using Student's *t*-test.

## 3. Results 

### 3.1. Changes in the Radon Concentration

Radon concentrations in the mouse cages are shown in Figures 1(b) and 1(c). The mean concentrations of background and treatment radon were approximately 20 Bq/m^3^ and 4000 Bq/m^3^, respectively.

### 3.2. Effects of Radon Inhalation on Hepatic Functions following Alcohol Administration

**Figure 2:**
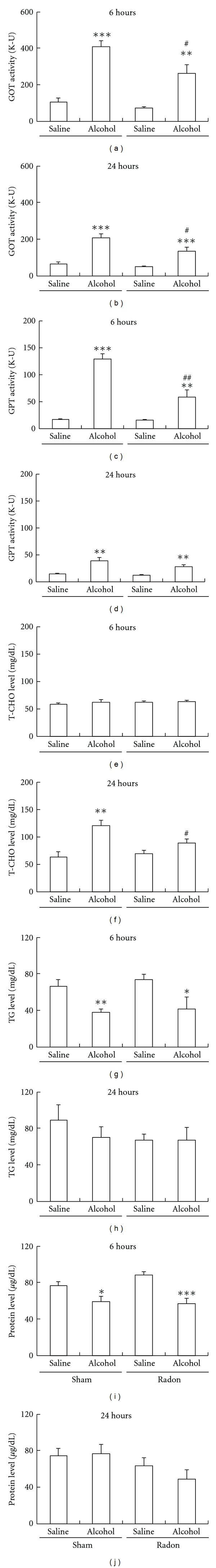
Effects of radon inhalation on hepatic function-associated parameters in the serum of alcohol-administrated mice. Each value indicates the mean ± SEM. The number of mice per experimental point is 4–6. *P < 0.05, **P < 0.01, ***P < 0.001 versus each saline administrated mice. ^#^ *P* < 0.05, ^##^ *P* < 0.01 versus sham-inhaled and alcohol-administrated mice.

To assess the effects of radon inhalation on alcohol-induced hepatopathy, various parameters of hepatic functions were assayed in serum following radon inhalation. First, it was assessed whether alcohol administration induces acute alcohol-induced hepatopathy. Six hours after alcohol administration in the absence of radon (sham), the activities of GOT and GPT in serum were significantly higher (*P* < 0.001), and the TG (*P* < 0.01) and protein level (*P* < 0.05) in serum were significantly lower than in saline-administrated mice. Twenty-four hours after alcohol administration in the absence of radon, the activities of GOT (*P* < 0.001), GPT (*P* < 0.01), and T-CHO (*P* < 0.01) in serum were significantly higher than in saline-administrated animals (Figure 2). Six hours after alcohol administration following radon inhalation, the activities of GOT and GPT in serum were significantly higher (*P* < 0.01) and the TG (*P* < 0.05) and protein (*P* < 0.001) in serum were significantly lower than in radon-inhaled and saline-administrated mice. Twenty-four hours after alcohol administration following radon inhalation, the activities of GOT (*P* < 0.001) and GPT (*P* < 0.01) in serum were significantly higher than in radon-inhaled and saline-administrated mice (Figure 2).

Next, we assessed whether radon inhalation inhibits acute alcohol-induced hepatopathy. In mice injected with alcohol following radon inhalation, GOT activities in serum were significantly lower (6 h or 24 h, *P* < 0.05) than in sham-inhaled and alcohol-administrated mice. Similarly, GPT activity (6 h, *P* < 0.01) and T-CHO (24 h, *P* < 0.05) in the serum of radon-inhaled mice were significantly lower than in sham-inhaled and alcohol-administrated mice (Figure 2).

### 3.3. Effects of Radon Inhalation on TG Level in Liver following Alcohol Administration

**Figure 3:**
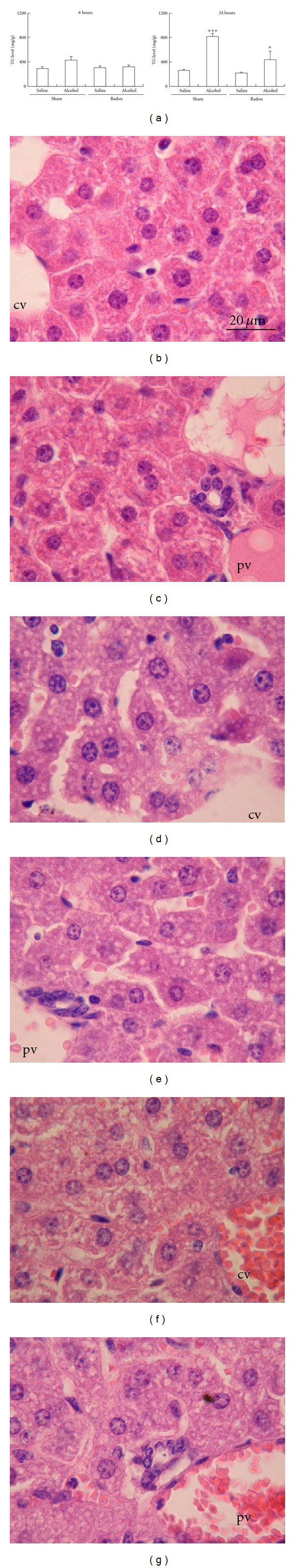
Effects of radon inhalation on TG level in liver (a) and histological changes in mouse liver after 24 hours following alcohol administration. (b), (c) sham inhalation before saline administration; (d), (e) sham inhalation before alcohol administration; (f), (g) radon inhalation before alcohol administration. The length of the scale bar is 20 *μ*m. All samples were stained with HE. The area of the vacuole formations surrounding the central vein (cv) and portal vein (pv). Each value indicates the mean ± SEM. The number of mice per experimental point is 4–6. *P < 0.05, ***P < 0.001 versus each saline-administrated mice.

To assess the effects of radon inhalation on alcohol-induced fatty liver, TG level in the liver was assayed following radon inhalation. First, we assessed whether alcohol administration induces fatty liver. Twenty-four hours after alcohol administration in the absence of radon, the TG level in the liver was significantly higher (*P* < 0.001) than in saline-administrated animals (Figure 3(a)). Twenty-four hours after alcohol administration following radon inhalation, the TG level in the liver was significantly higher (*P* < 0.05) than in radon-inhaled and saline-administrated mice (Figure 3(a)). 

Next, we assessed whether radon inhalation inhibits fatty liver. Twenty-four hours after alcohol administration, the TG level in the liver was 25% lower in radon-pretreated and alcohol-administrated animals than in sham-inhaled and alcohol-administrated animals, but this difference was not significant (Figure 3(a)).

### 3.4. Histological Observation in Livers following Alcohol Administration

 To reveal which part of the liver has the most fatty degeneration, hepatocytes were examined following alcohol administration. Centrilobular hepatocytes were vacuolated following alcohol administration (Figures 3(d) and 3(f)). Periportal hepatocytes were also vacuolated following alcohol administration, but the vacuole formation of centrilobular hepatocytes was greater than in periportal hepatocytes (Figures 3(e) and 3(g)). No vacuole formation was observed in hepatocytes in saline-administrated mice (Figures 3(b) and 3(c)).

### 3.5. Effects of Radon Inhalation on Oxidative Damage in Liver following Alcohol Administration

**Figure 4:**
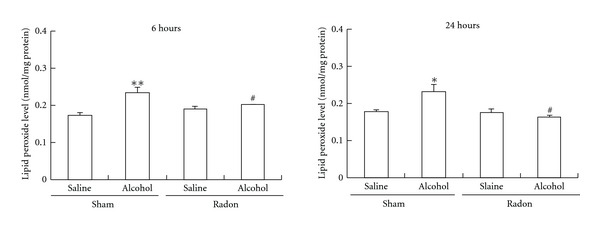
Effects of radon inhalation on lipid peroxide level in liver after alcohol administration. Each value indicates the mean ± SEM. The number of mice per experimental point is 5-6. *P < 0.05, **P < 0.01 versus each saline-administrated mice, ^#^ *P* < 0.05 versus sham inhaled and alcohol-administrated mice.

To assess the effects of radon inhalation on alcohol-induced oxidative damage, lipid peroxide level in the liver was assayed following radon inhalation. First, we assessed whether alcohol administration induces oxidative damage. Six or twenty-four hours after alcohol administration in the absence of radon, lipid peroxide in the liver was significantly higher (6 h or 24 h, *P* < 0.01 and *P* < 0.05, resp.) than in sham-inhaled and saline-administrated mice (Figure 4). 

Next, we assessed whether radon inhalation inhibits oxidative damage induced by alcohol administration. Six or twenty-four hours after alcohol administration following radon inhalation, lipid peroxide in the liver was significantly lower (*P* < 0.05) than in sham-inhaled and alcohol-administrated mice (Figure 4).

### 3.6. Effects of Radon Inhalation on Antioxidative Functions in Liver following Alcohol Administration

**Figure 5:**
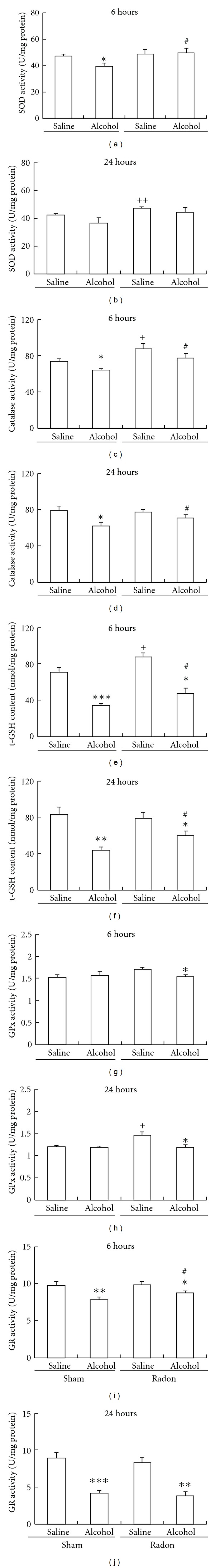
Effects of radon inhalation on antioxidative-associated parameters in mouse liver after alcohol administration. Each value indicates the mean ± SEM. The number of mice per experimental point was 5-6. *P < 0.05, **P < 0.01, and ***P < 0.001 versus each saline-administrated mice, ^#^ *P* < 0.05, versus sham inhaled and alcohol-administrated mice, and ^+^ *P* < 0.05, ^++^ *P* < 0.01 versus sham-inhaled and saline-administrated mice.

To clarify the mechanisms underlying the inhibitory effect of radon inhalation on acute alcohol-induced hepatopathy, antioxidant-associated substances such as SOD, catalase, t-GSH, GPx, and GR were examined. First, we assessed whether radon inhalation activates antioxidative functions in the liver. In mice injected with saline following radon inhalation, catalase activity and t-GSH content in the liver (6 h, *P* < 0.05) and the activities of SOD (24 h, *P* < 0.01) and GPx (24 h, *P* < 0.05) in the liver were significantly higher than in sham-inhaled and saline-administrated mice (Figure 5).

Next, we assessed whether alcohol administration depresses antioxidative functions in the liver. Six hours after alcohol administration in the absence of radon, the activities of SOD (*P* < 0.05), catalase (*P* < 0.05), and GR (*P* < 0.01) and t-GSH content (*P* < 0.001) in the liver were significantly lower than in sham-inhaled and saline-administrated mice. Similarly, 24 hours after alcohol administration in the absence of radon, the activities of catalase (*P* < 0.05) and GR (*P* < 0.001) and t-GSH content (*P* < 0.01) in the liver were significantly lower than in saline-administrated and sham-inhaled mice. Six hours after alcohol administration following radon inhalation, the activities of GPx and GR and t-GSH content in the liver were significantly lower (*P* < 0.05) than in radon-inhaled and saline-administrated mice. Similarly, 24 hours after alcohol administration following radon inhalation, the activities of GPx (*P* < 0.05) and GR (*P* < 0.01) and t-GSH content (*P* < 0.05) in the liver were significantly lower than in radon-inhaled and saline-administrated mice (Figure 5).

Furthermore, we assessed whether radon inhalation activates antioxidative functions following alcohol administration. Six hours after alcohol administration following radon inhalation, the activities of SOD, catalase, and GR and t-GSH content in the liver were significantly higher (*P* < 0.05) than in sham-inhaled and alcohol-administrated mice. Similarly, 24 hours after alcohol administration following radon inhalation, catalase activity (*P* < 0.05) and t-GSH content (*P* < 0.05) in the liver were significantly higher than in sham-inhaled and alcohol-administrated mice (Figure 5).

## 4. Discussion

Low-dose irradiation promotes a small induction of ROS *in vivo *and induces the production of antioxidant substances, including SOD, catalase and glutathione, in various organs [23–25]. Previous studies have shown that low-dose X-irradiation enhances antioxidative functions in the liver of mice [26] and reduces oxidative damage such as ischemia-reperfusion injury [27], cold injury [28], and liver damage [29–31]. It appears to be highly possible that low-dose X-irradiation helps to prevent or reduce ROS-related damage. 

Recently, we have reported that radon inhalation has an effect similar to X-irradiation. For example, radon inhalation activates antioxidative functions in many organs of mice and inhibits CCl_4_-induced liver and renal damage [18, 32, 33], type-1 diabetes [8], and pain [8, 34]. Interestingly, the absorbed dose of radon is much lower than X-irradiation. Our previous study suggested that radon inhalation at a concentration of 4000 Bq/m^3^ for 12 hours activated increased SOD activity in mouse liver [7]. According to Sakoda's report [12], the absorbed dose by the liver was approximately 300 nGy under experimental conditions; however, the absorbed dose does not include exposure to radiation from the radon progeny concentration. Therefore, our radon exposure system in the present study probably has a slightly higher absorbed dose by the lungs because the radon progeny concentration is uncontrolled.

Production of free radicals contributes to the development of alcohol-induced liver damage [14–17]. Alcohol administration has been found to cause the accumulation of ROS, including O_2_^−^, ^−^OH, and H_2_O_2_, and to increase lipid peroxide in the liver [16]. Alcohol administration leads to GSH depletion in the liver [35, 36], suggesting direct conjugation of GSH with acetaldehyde and reactive intermediates of alcohol oxidation. The results of this study show that not only t-GSH but also other antioxidant enzymes, such as SOD, catalase, and GR, decreased following alcohol administration. In addition, alcohol administration significantly increased lipid peroxide in the liver. These findings indicate that alcohol administration induced oxidative damage. Moreover, alcohol administration significantly increased GOT and GPT activities and T-CHO in serum and significantly decreased TG in serum. These findings suggest that alcohol administration induced hepatopathy. Furthermore, alcohol administration significantly increased TG in the liver, suggesting that alcohol administration induced fatty liver. 

Our previous study suggested that radon inhalation at a concentration of 2000 Bq/m^3^ for 24 hours significantly increased GPx activity and t-GSH content in mouse liver [18]. Another report suggested that activation of SOD activities induced by radon inhalation showed a dose-rate effect [7]. In fact, radon inhalation significantly increased catalase activity, t-GSH content, SOD activity, and GPx activity in the liver. These findings suggest that radon inhalation activated antioxidative functions in the liver. 

The results of this study show that the activities of GOT and GPT in serum and the levels of TG and lipid peroxide in the liver of radon-inhaled and alcohol-administrated mice were significantly lower than those of sham-inhaled and alcohol-administrated mice. These findings suggest that radon inhalation inhibited acute alcohol-induced hepatopathy, fatty liver, and oxidative damage. 

To clarify the mechanisms underlying the inhibitory effect of radon inhalation, we examined antioxidant-associated substances such as SOD, catalase, t-GSH, GPx and GR. Many studies have shown that administration of antioxidants agents can prevent alcohol-induced liver injury. For example, quercetin prevents ethanol-induced dyslipidemia and mitochondrial oxidative damage [37]. Another report suggested that *Emblica officinalis* prevents ethanol-induced hepatic injury by ameliorating oxidative stress [38]. In this study, antioxidative functions in the liver of radon-inhaled and alcohol-administrated mice were significantly higher than in sham-inhaled and alcohol-administrated mice. These findings suggest that radon inhalation inhibits acute alcohol-induced hepatopathy in mice due to the activation of antioxidative functions induced by radon inhalation.

## 5. Conclusion

 Radon inhalation activates antioxidative functions and inhibits not only acute alcohol-induced oxidative damage but also hepatopathy and fatty liver in mice. The activation of antioxidative functions induced by radon inhalation may detoxify ROS induced by alcohol metabolism. Radon therapy is performed for various diseases at Misasa Medical Center, Okayama University Hospital, Okayama Japan. Hepatic damage is not the main indication for radon therapy; however, our study strongly suggests that radon therapy is useful in the prevention of alcohol-induced hepatopathy.
